# Myeloid-derived PD-L1 characterizes spatially organized immune architecture in colorectal cancer

**DOI:** 10.3389/fimmu.2026.1763068

**Published:** 2026-02-06

**Authors:** Qian Wu, Stephanie Tissot, Allyson Peddle, Ke Yin, Xavier Sagaert, Gert De Hertogh, Benyagoub Abdelkader, Ting Pu, Filip Van Herpe, André D’Hoore, Sylvie Rusakiewicz, Sara Verbandt, Sabine Tejpar, Gertjan Rasschaert

**Affiliations:** 1 Digestive Oncology, Department of Oncology, KU Leuven, Leuven, Belgium; 2 Immune Landscape Laboratory, Centre Thérapies Expérimentales (CTE), Centre Hospitalier Universitaire Vaudois (CHUV), Lausanne, Switzerland; 3 Department of Pathology, University Hospitals Leuven and KU Leuven, Leuven, Belgium; 4 Gastrointestinal Oncology Department, University Hospitals Leuven, Leuven, Belgium; 5 Abdominal Surgery Department, University Hospitals Leuven, Leuven, Belgium

**Keywords:** CD8 T lymphocytes, colorectal cancer, immune checkpoint inhibitors, immunotherapy, multiplex immunofluorescence, PD-L1

## Abstract

**Introduction:**

The tumor immune microenvironment (TIME) is highly heterogeneous and strongly influences immunotherapy outcomes and patient prognosis in colorectal cancer (CRC). In this exploratory study, we used three multiplex immunofluorescence (mIF) assays to characterize the spatial immune microenvironment associated with high CD8/PD-L1 infiltration.

**Methods:**

Three mIF assays quantified the cell densities (cells/mm^2^) of CD3, CD8, PD-L1, PD-1, CD163, CD56, CD4, Foxp3, Granzyme B (GrzB), CD20, CD11c, CD15, Ki67, and cytokeratin (CK) in the invasive margin (IM) and tumor center (TC) using digital image analysis. Patients were stratified based on CD8/PD-L1 densities and their proximity (cut-off: 20μm) in IM and TC. Immune microenvironment composition was compared between high and low infiltration groups across IM and TC.

**Results:**

PD-L1 expression was predominantly driven from stromal and immune cells with enrichment at IM versus TC, particularly on CD163^+^ macrophages. Patients with high CD8/PD-L1 infiltration demonstrated significantly increased densities of CD20^+^, CD3^+^, PD-1^+^, CD8^+^PD-1^+^, and CD56^+^ natural killer (NK) cells across tumor tissue, specifically enriched at IM. CD4^+^Foxp3^+^ regulatory cells positively correlate with PD-1^+^, CD8^+^PD-1^+^, and CD56^+^ cells in IM but not TC.

**Conclusions:**

This exploratory mIF analysis identifies PD-L1 expression predominantly on stromal and immune cells, enriched in IM, particularly on CD163^+^ macrophages. High CD8/PD-L1 tumors display spatially organized IM-specific immune niches featuring coordinated effector-regulatory interactions. Comprehensive spatial profiling of IM-enriched populations, including B cells, CD163^+^ macrophages, regulatory T cells, and NK cells alongside CD8/PD-L1, may refine patient stratification for immunotherapy in CRC.

## 1 Introduction

Colorectal cancer (CRC) ranks third in global malignancies and remains a leading cause of cancer death (1). Although immune checkpoint inhibitors (ICIs) achieve durable benefit in mismatch repair-deficient/microsatellite instability-high (dMMR/MSI-H) tumors, most mismatch repair-proficient/microsatellite stable (pMMR/MSS) patients remain refractory (2–4). Emerging evidence of benefits in a fraction of pMMR/MSS cases shows that mismatch repair (MMR) status alone does not suffice to capture the immunologic heterogeneity of CRC (2, 5). These observations suggest that a more discriminating immune-based classification is needed.

Prognosis and therapeutic responsiveness in CRC are tightly linked to the tumor immune microenvironment (TIME) (6). Measures such as tumor-infiltrating lymphocytes (TILs) and the Immunoscore retain prognostic value independent of tumor-node-metastasis (TNM) stage, MMR status, and oncogenic drivers (7–9). By contrast, their utility in predicting response to ICIs remains limited. Despite the central role of PD-1/PD-L1 axis in immunotherapy, PD-L1 alone has shown inconsistent predictive value in metastatic CRC, including dMMR/MSI-H cases (9, 10). These limitations highlight the urgent need of the development of composite biomarkers that integrate both effector and suppressive immune features within the TIME.

To address this need, the Immunoscore-Immune Checkpoint (Immunoscore-IC) model was developed and prospectively validated in the AtezoTRIBE trial (11). Immunoscore−IC integrates CD8^+^ infiltration, PD−L1^+^ cell density and CD8/PD−L1 spatial proximity in the tumor center (TC), and shows superior performance over standard immune readouts for predicting benefit from ICIs in both dMMR/MSI−H and pMMR/MSS settings (9). Its predictive value was also confirmed in non–small cell lung cancer (NSCLC), supporting its broad applicability as an immune classification framework (12). Nevertheless, the underlying TIME contexture of Immunoscore-IC has not been well defined, particularly regarding the regional distribution and cellular composition of CD8^+^ and PD-L1^+^ populations across tumor compartments, and the broader immune contexture associated with high CD8/PD-L1 infiltration.

In this exploratory study, we used three multiplex immunofluorescence (mIF) panels designed and stained by the IMMU can consortium to comprehensively characterize the spatial immune architecture underlying high CD8/PD-L1 infiltration in CRC. By quantifying immune cell densities across both the invasive margin (IM) and TC, we identified several promising spatial patterns. PD-L1 expression mainly came from stromal and immune cells, enriched in IM, particularly on macrophages (CD163^+^). High CD8/PD-L1 tumors harbor spatially organized IM-specific niches featuring coordinated effector populations (CD8^+^/CD3^+^ T cells, CD20^+^ B cells, CD56^+^ natural killer (NK) cells) and regulatory populations (PD-L1^+^ cells, PD-1^+^ and CD8^+^PD-1^+^ checkpoint-expressing T cells). Notably, CD4^+^Foxp3^+^ regulatory T (Treg) cells demonstrated IM-specific positive correlations with PD-1^+^, CD8^+^PD-1^+^, and CD56^+^ NK cells. These observations provide support for an IM-specific immune architecture that refines current immune classifications and may also aid in the stratification of patients who may elicit an improved response to ICIs in CRC.

## 2 Materials and methods

### 2.1 Patient characteristics

This study analyzed patient samples from the IMMUcan project, a large-scale European consortium investigating immune contexture and tumor microenvironment in CRC. This study was designed as an exploratory analysis to investigate spatial patterns of immune cell organization in CRC using high-dimensional mIF. Twenty patients with primary CRC surgery were included. This IMMUcan study was approved by the UZ Leuven Medical Ethical Committee (S61000) and all patients provided written informed consent. Twenty patients (9 MSI-H and 11 MSS) with primary CRC who underwent surgical resection at University Hospitals Leuven were included. All patients were treatment-naïve (no prior neoadjuvant chemotherapy or radiotherapy). Of 20 patients enrolled, all underwent mIF staining. Based on staining quality, 14 mIF-stained samples (7 MSI-H, 7 MSS) were suitable for analysis with Panel 1, and 15 samples (8 MSI-H, 7 MSS) were analyzed with Panels 2 and 3. Samples with suboptimal staining quality were excluded from quantitative analysis. Clinical characteristics of 15 patients were included in **Supplementary Table S1**. The cohort included 6 males and 9 females.

### 2.2 Multiplexed immunofluorescence staining

**Figure 1:**
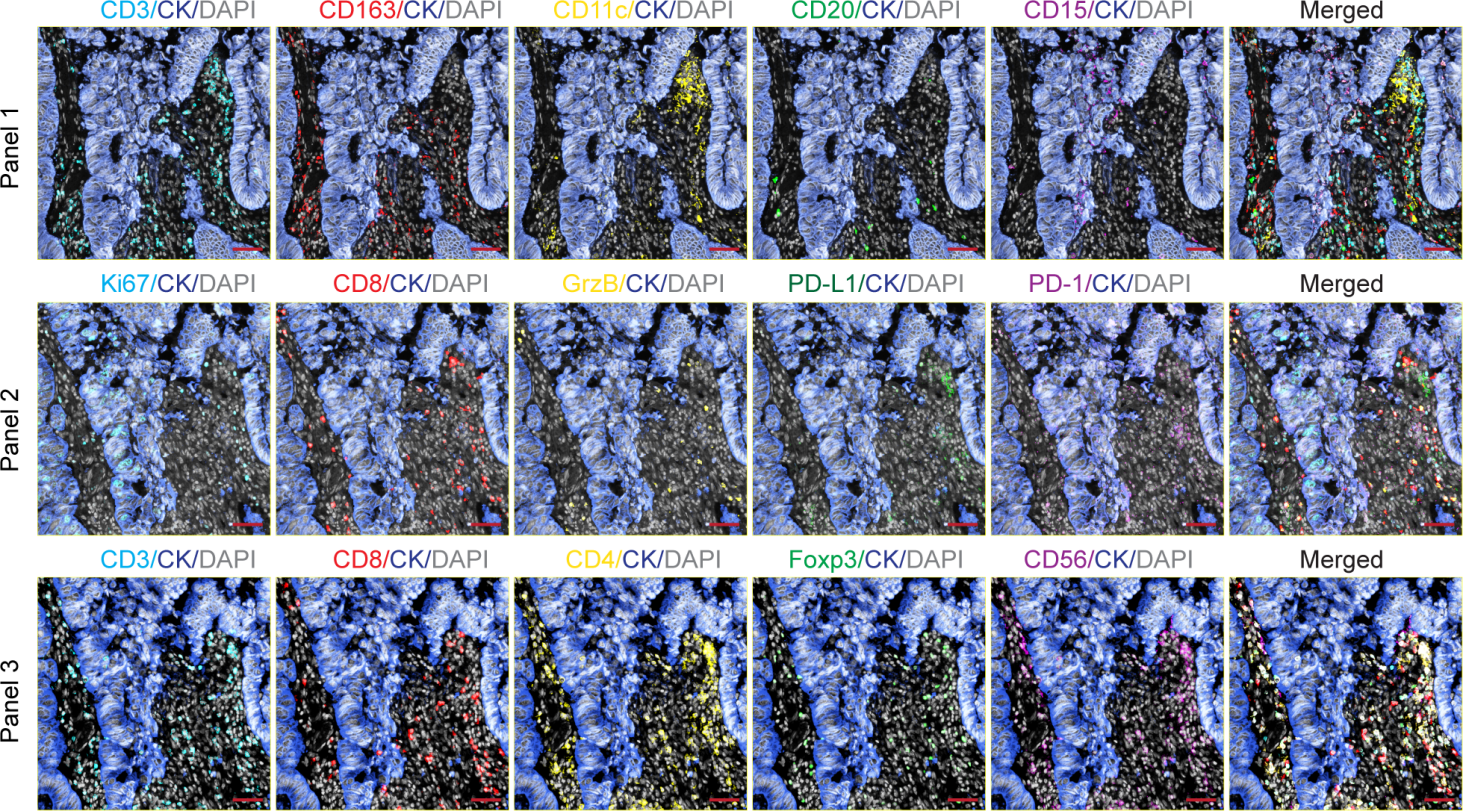
mIF profiling of immune cells in CRC. Representative mIF images of serial tissue sections stained with three different antibody panels. Each panel highlights distinct immune cell markers and epithelial tumor regions. Scale bar: 50 μm.

Formalin-fixed paraffin-embedded (FFPE) sections (4 μm) from primary CRC specimens were subjected to mIF. Tumor-enriched regions were identified by two independent pathologists and only slides meeting predefined quality control criteria were included (13). In the IMMUcan cohort, three biomarker panels were applied: Panel 1 characterized overall immune composition, while Panel 2 and Panel 3 focused on T-cell subsets and cytotoxic activity (**Figure 1**). Markers included CK, CD20, CD11c, CD163, CD15, CD56, CD3, CD4, CD8, Foxp3, GrzB, Ki67, PD-1, and PD-L1. All slides (mIF and Hematoxylin &Eosin (H&E) slides) were scanned using the PhenoImager™ HT (Akoya) with MOTiF™ whole-slide multispectral imaging. Images were analyzed using QuPath 0.4.3 (RRID: SCR_018257) with consistent thresholds for signal intensity, cell size, and nuclear morphology. Detailed protocols are available from the authors upon request.

### 2.3 mIF reagents

Multiplexed staining was performed on CRC tissue sections on automated Ventana Discovery Ultra staining module (ROCHE). Slides were placed on the staining module for deparaffinization, epitope retrieval (64 minutes at 98 °C) and endogenous peroxidase quenching (Discovery Inhibitor, 8 minutes, Ventana). Each round of staining included non-specific site blocking (Discovery Goat IgG and Discovery Inhibitor, Ventana), primary antibody incubation and secondary HRP-labeled antibody incubation for 16 minutes with Discovery OmniMap HRP (Roche, cat#760-4311, RRID: AB_2811043 and Roche, cat# 760-4310, RRID: AB_2885182). Covalent dye labeling was then performed using the OPAL™ reactive fluorophore detection (Akoya Biosciences, Marlborough, MS, USA) for 12 min followed by subsequent heat denaturation of the antibodies for a next round of staining. The following primary antibodies were performed: anti-human Cytokeratin (Agilent, cat#GA053, RRID: AB_2892089); anti-human CD20cy (Agilent, cat#M0755, RRID: AB_2282030); anti-human CD15 (BioLegend, cat# 301902, RRID: AB_314194); anti-human CD11c (Cell Marque, cat#111M, RRID: AB_3714915); anti-human CD163 (Diagnostics BioSystems, cat# Mob460, RRID: AB_3714914); anti-human CD56 (Cell Marque, cat# 156R-9, RRID: AB_2941091); anti-human CD3 (Agilent, cat#A0452, RRID: AB_2335677); anti-human CD4 (Cell Marque, cat#104R, RRID: AB_1516770); anti-human CD8 (Cell Marque, cat#108R, RRID: AB_2892088); anti-human FoxP3 (Abcan, cat#ab99963, RRID: AB_10675258); anti-Granzyme B (Monosan, cat# MON7029C, clone GrB-7); anti-human Ki67 (Cell Marque, cat#275R-16, RRID: AB_1158037); anti-human PD1 (Biocare Medical, cat#ACI3137, RRID: AB_2566065); anti-human PD-L1 (Cell Signaling Technology, cat#13684, RRID: AB_2687655) were used. Nuclei were visualized by final manual incubation with Spectral DAPI (1/10, FP1490, Akoya Biosciences) for 20 minutes. Multiplex IF images were acquired on PhenoImager HT 2.0, allowing a whole slide multispectral imaging acquisition followed by unmixing (Akoya Biosciences). IF signal extractions from qptiff were performed using QuPath, enabling a per-cell analysis of IF markers of multiplex-stained tissue sections and counting of every cell population. The slides were mounted with fluorescence mounting medium (Dako, ref#S3023) using the glass coverslip and stored in the dark at 4 °C until scanned within 24 hours.

### 2.4 Hematoxylin and eosin staining

Tissue slides were incubated at 60 °C for 30 minutes, followed by deparaffinization in xylene (3 min ×2, 5 min ×1) and rehydration through graded ethanol (100% ×3, 96%, and 70%, each for 1 min). Slides were rinsed under tap water for 5 minutes, then stained with hematoxylin for 5–10 minutes. After a 20-second rinse in warm water, differentiation was performed in acid alcohol for 2 seconds, followed by bluing under warm tap water until nuclei appeared distinctly blue. Slides were dipped in 70% ethanol (15 sec), counterstained with eosin (5 sec), and dehydrated through 96% ethanol (2 × 15 sec) and 100% ethanol (2 × 15 sec, 1 × 30 sec). Clearing was conducted in xylene (2 × 15 sec, 1 × 1 min). Slides were mounted with Eukit and scanned using the Nanozoomer S60 (Hamamatsu).

### 2.5 Region selection and image analysis

Regions of interest (ROIs) were defined based on matched hematoxylin and eosin (H&E)-stained slides and subsequently aligned with serial mIF sections for quantitative analysis (**Supplementary Figure S1A**). The TC and IM were delineated, with the IM defined as the 1 mm-wide zone spanning the tumor–stroma interface (14). For each case, up to 5 representative fields (1000 mm × 1000 mm) from both TC and IM were analyzed (**Supplementary Figure S1B**). Image analysis was performed in QuPath, applying uniform thresholds for signal intensity, cell size, and nuclear morphology to ensure analytical consistency across all slides.

### 2.6 Immune-based signatures

The Immunoscore was calculated based on CD3^+^ and CD8^+^ densities in TC and IM, dichotomized at the 50th percentile. The score ranged from 0 to 4 (I0–I4) and was classified as low (0–2) and high (3–4) (15). In parallel, the CD8/PD-L1 signature captured the local immune architecture by integrating CD8^+^ densities, PD-L1^+^ densities, and their spatial proximity within 20 μm of each other (11). Spatial proximity was defined as CD8^+^ and PD-L1^+^ cells located within 20 μm of each other in custom R scripts (R version 4.4.0, R Core Team, 2024, RRID: SCR_001905). This metric was dichotomized at the median, yielding scores of 0-6, and categorized as low (0–4) and high (5–6). The high/low CD8–PD-L1 feature was used for descriptive analyses to explore immune patterns associated with Immunoscore-IC parameters.

### 2.7 Statistical analysis

This was a retrospective observational study without randomization. Sample size was determined by tissue availability. All statistical analyses were performed using GraphPad Prism 10 (RRID: SCR_002798). Clinical and molecular features were compared using Wilcoxon, Chi-square, Fisher’s exact, or Mann–Whitney U tests, as appropriate. Correlation analyses were performed using linear regression and Pearson correlation. Statistical significance was defined as p ≤ 0.05 for all comparisons.

## 3 Results

### 3.1 PD-L1 expression is predominantly stromal and myeloid derived in CRC

**Figure 2:**
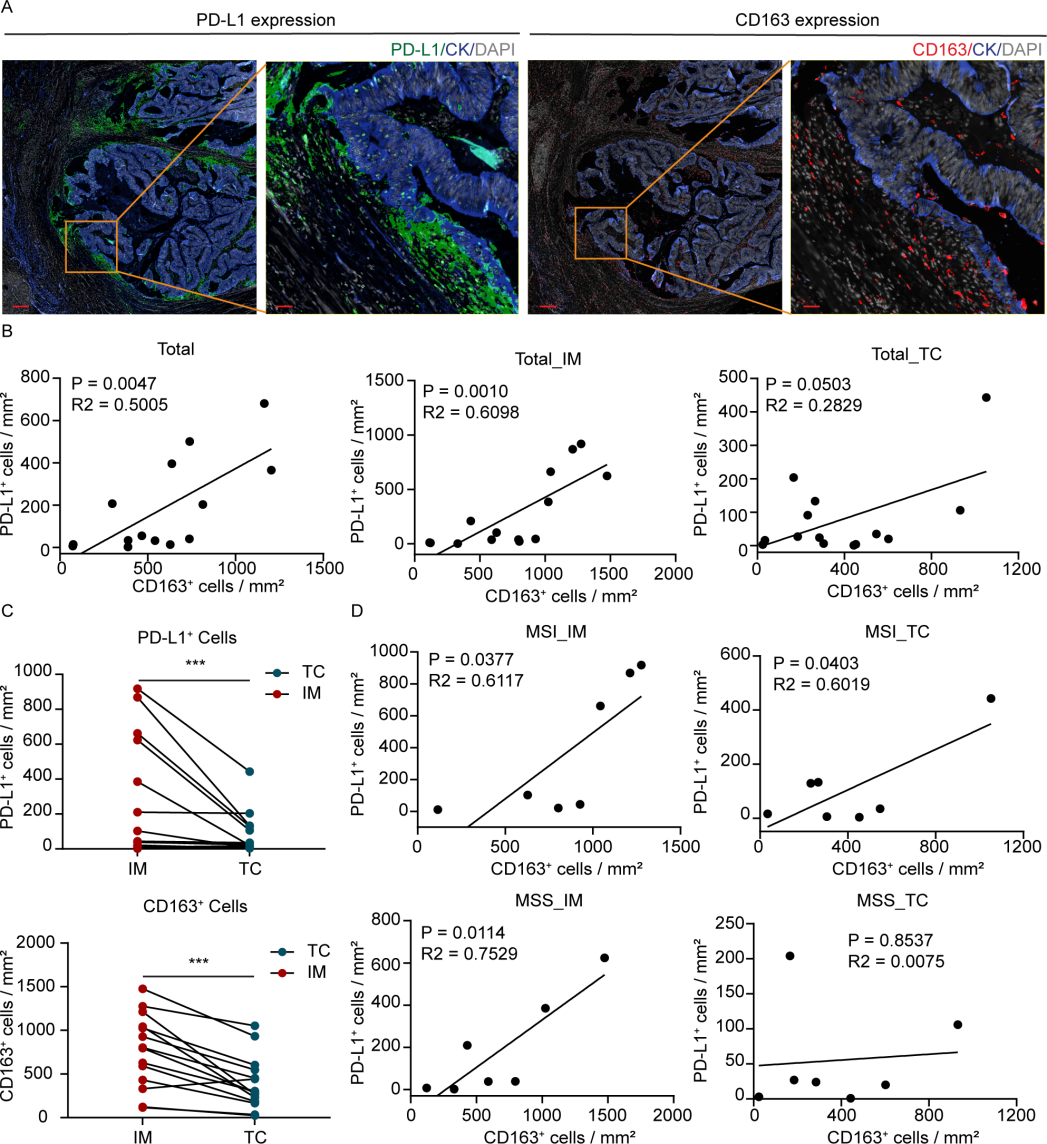
Spatial enrichment of PD-L1^+^ and CD163^+^ myeloid cells in CRC. **(A)** Representative mIF images showing PD-L1 and CD163 expression in CRC tissues. Scale bar: 250 μm (left); 100 μm (right). **(B)** Correlation between PD-L1^+^ and CD163^+^ cell infiltration across whole tumor regions in 14 CRC patients. **(C)** Paired comparison of PD-L1^+^ and CD163^+^ cell densities between IM and TC using the Wilcoxon matched-pairs signed-rank test (n=14). **(D)** Correlation between PD-L1^+^ and CD163^+^ cell densities in CRC with different microsatellite statuses, stratified by tumor location (IM or TC). ns, *p* > 0.05; ***p < 0.001.

Unlike the tumor-centric PD-L1 pattern of NSCLC, mIF analysis of CRC specimens revealed a distinct spatial distribution of PD-L1 in CRC (**Supplementary Figure S2A**). As seen in Panel 2, the expression of PD-L1 was predominantly localized to stroma and immune (CK^-^) cells, not in epithelial tumor (CK^+^) cells and the statistical analysis confirmed this (*p* = 0.0001) (**Supplementary Figures S2B-D**)., indicating that the primary source of PD-L1 in CRC is derived from non-malignant cellular compartments Spatial mapping of sequential mIF panels demonstrated the enriched clustering and proximity of PD-L1^+^ cells and CD163^+^ macrophages at the tumor–stromal interface (**Figure 2A**). Linear regression revealed a significant positive association between PD-L1^+^ and CD163^+^ cell densities across different tumor compartments, with the strongest correlation specifically in the invasive margin, suggesting a compartment-specific spatial organization of this myeloid-derived checkpoint axis (**Figure 2B**). Quantitative analysis of cell densities confirmed statistically significant enrichment of both PD-L1^+^ and CD163^+^ cells in the IM compared to the TC (*p* = 0.0002 and *p* = 0.0006, respectively) (**Figure 2C**), validating the spatial compartmentalization of this myeloid-derived checkpoint axis. Furthermore, subgroup analysis showed that MSI-H CRC showed a positive correlation between PD-L1^+^ and CD163^+^ cells in both IM and TC regions, whereas in MSS CRC, this correlation was observed only in the IM region (**Figure 2D**). The non-tumor-centric pattern of PD-L1 expression in CD163^+^ macrophages represents a distinctive myeloid-derived spatial organization. And the IM-enriched distribution defines a spatially organized immunoregulatory interface that may influence.

### 3.2 CD8/PD-L1 infiltration represents distinct immune axes

**Figure 3:**
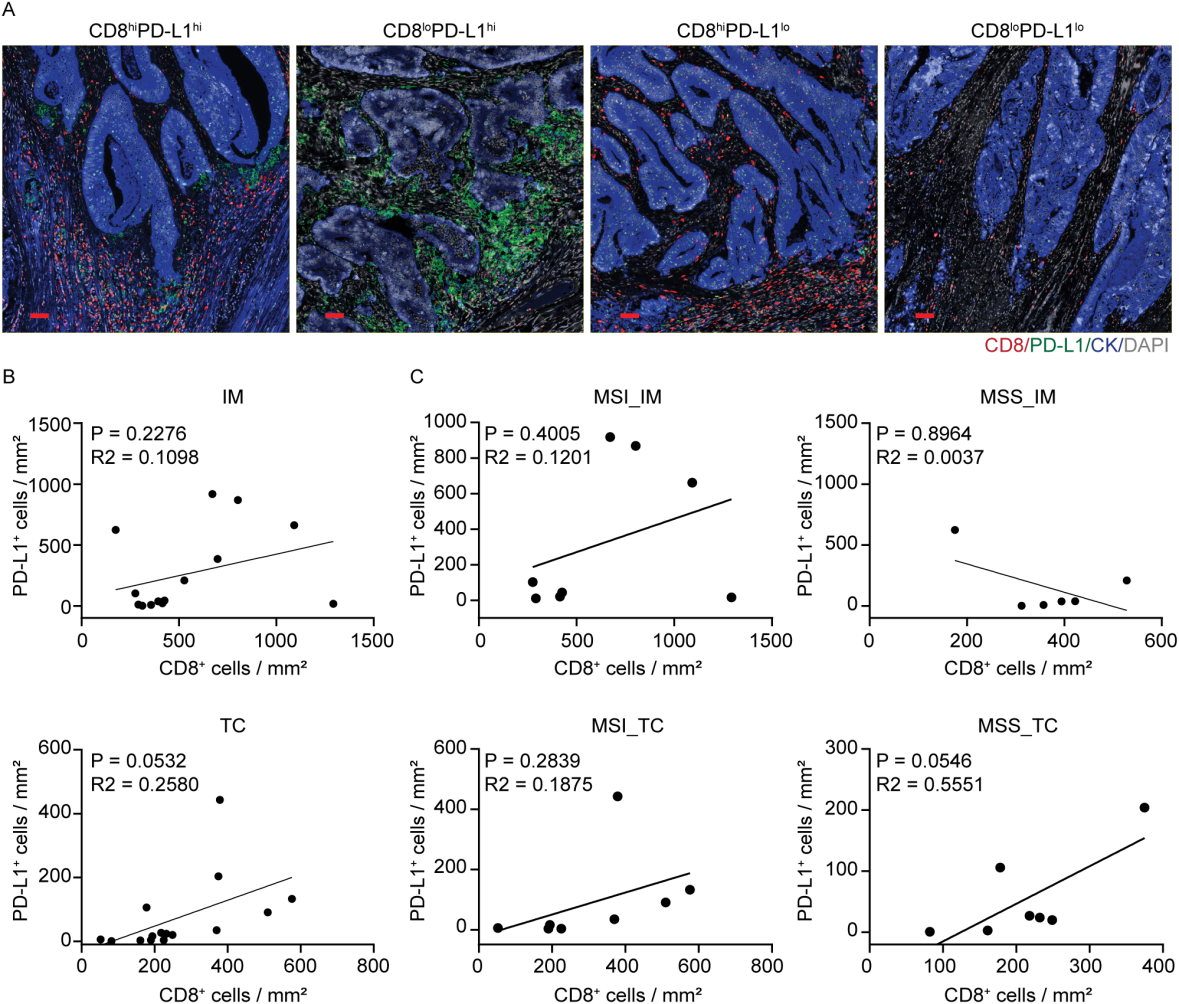
Spatial distribution and correlation of CD8^+^ and PD-L1^+^ cells in CRC. **(A)** Four representative expression patterns of CD8 and PD-L1 in IM and TC. Scale bar: 100 μm. **(B)** Correlation between CD8^+^ and PD-L1^+^ cell densities across tumor regions (IM and TC) in CRC tissues. **(C)** Correlation between CD8^+^ and PD-L1^+^ cell densities in CRC with different microsatellite statuses, stratified by tumor location (IM and TC).

Building on the Immunoscore-IC framework (9, 11) and enrichment in PD-L1 expression at the IM observed above in CD163^+^ myeloid cells, we analyze the spatial relationship between CD8^+^ and PD-L1^+^ cells in both IM and TC for 15 primary CRC samples. Spatial mapping pinpointed four distribution patterns for CD8^+^ and PD-L1^+^ cells in CRC (**Figure 3A**). Correlation analysis revealed no significant correlation between CD8^+^ and PD-L1^+^ cells in either region, with similar patterns observed in MSS and MSI-H subgroups (**Figures 3B, C**). These results suggest that the infiltration of CD8^+^ T-cell and PD-L1^+^ cell concentrations reveal different immune axes, one reflective of cytotoxic effector activity, and the other of myeloid-mediated immunoregulation.

To further define the immune context in which Immunoscore-IC predictive contribution applies, we measured CD8^+^ and PD-L1^+^ cell densities together with their spatial proximity in both IM and TC to classify tumors into high and low CD8/PD-L1 infiltration groups (**Supplementary Figure S1B**). Six cases were classified as high CD8/PD-L1 infiltration (5 MSI-H and 1 MSS), and nine cases were assigned as low infiltration (3 MSI-H and 6 MSS). This distribution demonstrates that high CD8/PD-L1 infiltration does not occur only in MSI-H tumors, but in some MSS tumors, which suggests that this combined infiltration of CD8/PD-L1 might well represent different immune phenotypes apart from MMR status.

Collectively, although CD8^+^ and PD-L1^+^ cell infiltration represents distinct immune axes, their spatial distribution and proximity across tissue compartments creates a comprehensive understanding of high CD8/PD-L1 tumors and their spatially organized TIME.

### 3.3 CD8/PD-L1 spatial profiling reveals effector and suppressive features

In order to characterize the immune context associated with high CD8/PD-L1 infiltration, immune subsets were profiled across three mIF panels to characterize the immune context with regard to high CD8/PD-L1 infiltration. In Panel 1, CD20^+^ B cells were found to be enriched in the high CD8/PD-L1 group (*p* = 0.0220); however, no association was observed between these cells and CD8^+^ or PD-L1^+^ cells (**Table 1**). As the stained results in Panel 3 are consistent, CD3^+^ cells were also enriched in high CD8/PD-L1 tumors (*p* = 0.0127) and demonstrated a positive correlation with CD8^+^ T cells (*p* = 0.0067), indicating the co-infiltration of T-cell subsets. This finding is consistent with prior observations (16). CD163^+^ cells exhibited a significant correlation with PD-L1^+^ cells (*p* = 0.0047), consistent with the findings described above. Subgroup analysis showed that B cells (CD20^+^), which are the main component of tertiary lymphoid structures (TLSs), were significantly enriched in the IM within the high CD8/PD-L1 group (*p* = 0.0200) (**Supplementary Figure S3A**), consistent with IM-predominant TLS formation (17–19). In contrast, B cells showed no significant difference in the TC between high and low infiltration groups (p = 0.0869).

**Table 1:** Associations of immune cell subsets with CD8^+^ and PD-L1^+^ cells in CRC.

Cell types	High vs low CD8/PD-L1 (*p* value)	Correlation with CD8 cells (*p* value)	Correlation with PD-L1 cells (*p* value)
Panel 1. B, DC, neutrophils and macrophages			
CD20	*	ns	ns
CD11c	ns	ns	ns
CD15	ns	ns	ns
CD3	*	**	ns
CD163	ns	ns	**
Panel 2. cytotoxic and functional markers			
PD-1	**	**	***
CD8^+^PD-1^+^	**	***	**
Ki67	ns	ns	ns
GrzB	ns	ns	ns
Panel 3. T cell subset and NK cells			
CD3	*	***	ns
CD4	ns	*	ns
CD4^+^Foxp3^+^	ns	ns	*
CD56	*	**	ns

Statistical significance was determined using Spearman correlation or the Mann-Whitney U test, as appropriate. ns, p > 0.05; *p ≤ 0.05; **p < 0.01; ***p < 0.001.

**Figure 4:**
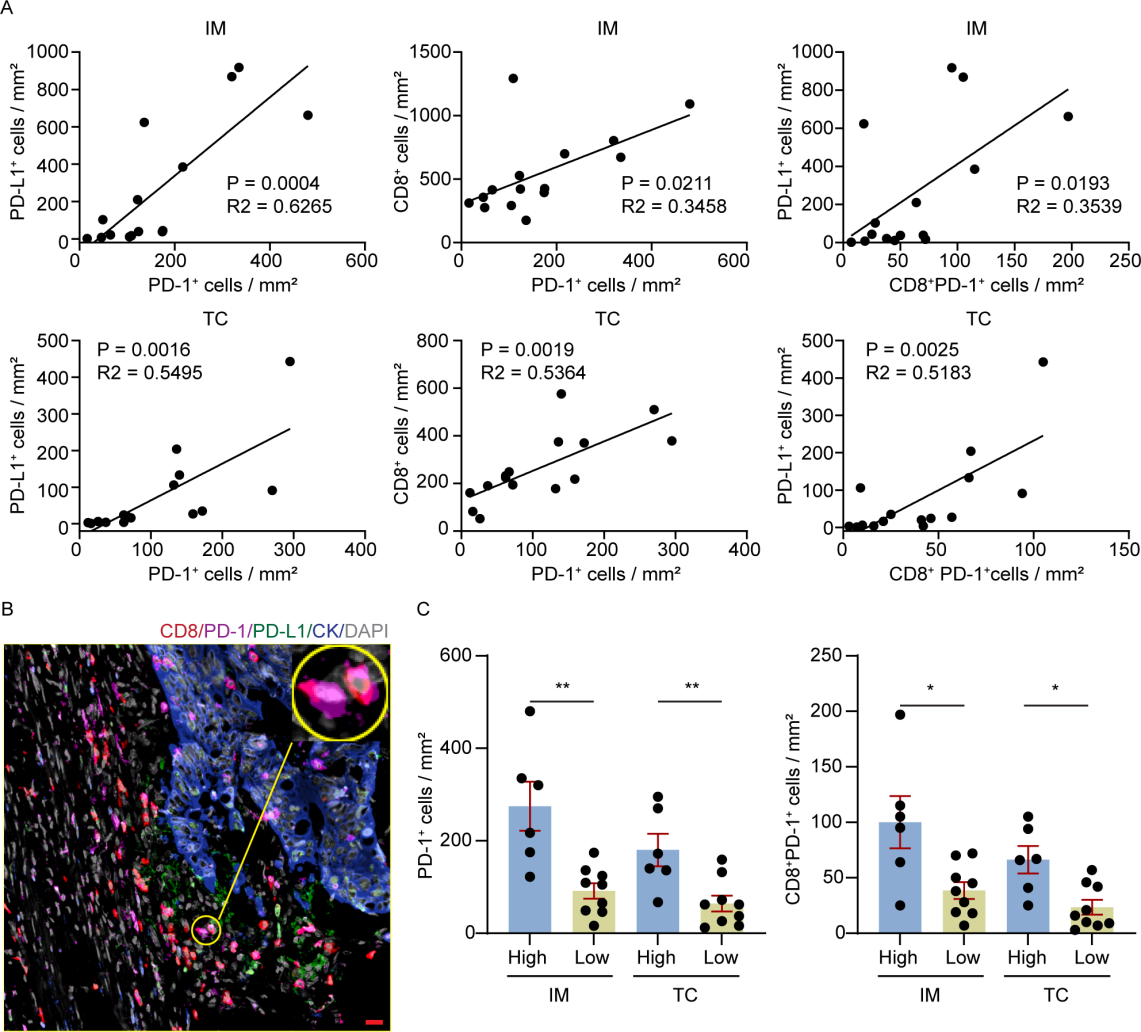
Association of PD-1^+^ and CD8^+^PD-1^+^ subsets in the immune microenvironment. **(A)** Correlation between CD8^+^ and PD-L1^+^ cell densities in CRC with different regions (IM and TC) **(B)** Representative showing co-localization of CD8 and PD-1 expression. Scale bar: 100 μm. **(C)** Comparison of PD-1^+^ and CD8^+^PD-1^+^ cell densities in IM and TC across CD8/PD-L1 infiltration groups. ns, *p* > 0.05; *p ≤ 0.05; **p < 0.01.

In Panel 2, PD-1^+^ and CD8^+^PD-1^+^ cells were both significantly enriched in the high CD8/PD-L1 group cells (*p* = 0.0028 and *p* = 0.0076, respectively) and showed significant positive correlations with CD8^+^ and PD-L1^+^ (**Table 1**). Subgroup analysis revealed that PD-1^+^ and CD8^+^PD-1^+^ cells displayed significant positive correlations with PD-L1^+^ cells and CD8^+^ cells in the IM and TC, affirming their function as an essential immunological link between PD-L1^+^ cells and CD8^+^ T cells (**Figure 4A**; **Supplementary Figure S3B**). In addition, both populations were enriched in the IM compared to the TC expression (*p* = 0.0006 and *p* = 0.0046, respectively) and a representative mIF image showed CD8^+^PD-1^+^ T cells in the IM co-existed with PD-L1 (**Supplementary Figure S3C****;****Figure 4B**). Furthermore, both populations were significantly higher across both IM (PD-1^+^ cell : *p* = 0.0028; CD8^+^PD-1^+^ cell : *p* = 0.0256) and TC (PD-1^+^ cell : *p* = 0.0070; CD8^+^PD-1^+^ cell : *p* = 0.0120) within high CD8/PD-L1 group compared to the low group (**Figure 4C**). This spatial association suggests that PD-1^+^ cells, in particular CD8^+^PD-1^+^ cells, may act as key intermediaries linking cytotoxic T cells to PD-L1-associated immunosuppressive niches within the TIME, at the level of spatial organization. These observations are consistent with those of Lin et al. (20), who reported that T−cell suppression in CRC is more closely related to the spatial proximity between PD−L1^+^ and PD−1^+^ cells than to the overall frequency of PD−L1^+^ cells. In this context, T−cell inhibition can be operationally characterized by direct PD−L1–PD−1 spatial contact rather than by PD−L1^+^ cell abundance alone, while recognizing that this reflects spatial proximity patterns. These findings collectively suggest that high CD8/PD-L1 tumors generate parallel-localized effector populations (CD3^+^, CD8^+^, CD20^+^) with enriched PD-L1^+^ myeloid cells and checkpoint-expressing cells (PD-1^+^, CD8^+^PD-1^+^ cells), forming an effector-immunoregulatory microenvironment in which PD-1/PD-L1 mediated immunosuppressive interactions are facilitated.

### 3.4 NK and T cell co-infiltration in the IM

In Panel 3, CD3^+^ and CD56^+^ NK cells were significantly enriched in the high CD8/PD-L1 group (*p* = 0.0360 and *p* = 0.0176, respectively) (**Table 1**). In addition, CD3^+^, CD4^+^ and CD56^+^ cell densities were positively correlated with CD8^+^ cells (*p* = 0.0015, *p* = 0.0104 and *p* = 0.0090, respectively), suggesting coordinated infiltration of multiple immune subsets. CD4^+^Foxp3^+^ regulatory T (Treg) cells showed a significant positive association with PD-L1^+^ cells (*p* = 0.0328), indicating an immunoregulatory component within the high PD-L1^+^ cells. We assessed the spatial relationship between Treg infiltration and PD-1^+^/CD8^+^PD-1^+^ cells, finding a significant positive correlation specifically in the IM but not TC (**Supplementary Figure S3D**). This IM-specific spatial relationship highlights the compartment-restricted organization of regulatory populations and their spatial proximity to checkpoint-expressing cells at the tumor-stromal interface.

**Figure 5:**
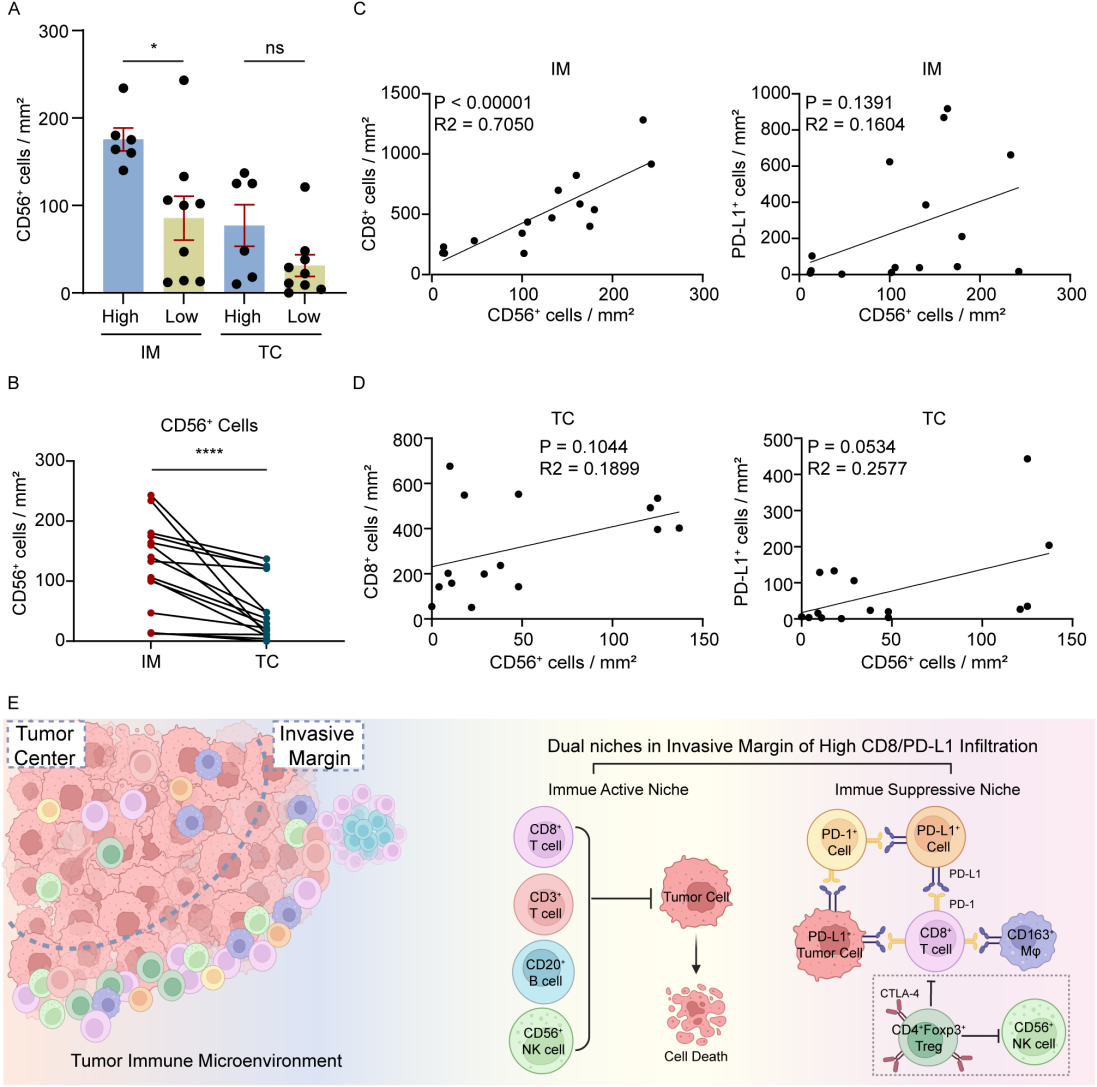
Region-specific infiltration of CD56^+^ NK cells with CD8^+^ T cells. **(A)** CD56^+^ NK cell densities in IM and TC across CD8/PD-L1 infiltration groups. **(B)** Paired comparison of CD56^+^ NK cell densities between IM and TC using the Wilcoxon matched-pairs signed-rank test (n = 15) **(C, D)** Correlations between CD56^+^ NK cell densities and CD8^+^ or PD-L1^+^ cell densities in CRC tissues with different regions (IM and TC*).***(E)** Illustration of the TIME across tumor regions (IM and TC) and the immune niches enriched in the IM with high CD8/PD-L1 infiltration. Dashed square indicated CD4^+^Foxp3^+^ Treg cells within potential regulatory function, associated with PD-L1^+^ cells and CD56^+^ NK cell infiltration that were not captured within the high CD8/PD-L1 infiltration classification. Treg, regulatory T; NK, natural killer; Mφ, macrophages. ns, *p* > 0.05; *p ≤ 0.05; ***p < 0.001.

CD56^+^ NK cells in IM were significantly more abundant in the high CD8/PD-L1 group than in low group but NK cells in the TC did not differ between low and high CD8/PD-L1 groups (*p* = 0.0120 and *p* = 0.0939, respectively) (**Figure 5A**). CD56^+^ NK cells showed significant enrichment in the IM relative to the TC by Wilcoxon testing (*p* < 0.0001) (**Figure 5B**). In the IM, CD56^+^ NK cells positively correlated with CD8^+^ cells but not with PD-L1^+^ cells, and no significant correlations were observed between CD56^+^ NK cells and either CD8^+^ or PD-L1^+^ cells in the TC (**Figures 5C, D**). These findings demonstrate IM-specific NK-cell enrichment and their spatial association with CD8^+^ cytotoxic T cells.

To investigate potential functional interactions between immune subgroups, spatial correlations were observed between NK cells, Treg cells, and checkpoint-expressing cells (PD-1^+^ and CD8^+^PD-1^+^). Within the IM, NK cells showed significant positive correlations with Treg cells and with PD-1^+^ and CD8^+^PD-1^+^ cells (**Supplementary Figure S3E**). These data reveal that CD56^+^ NK cells contribute to the IM-enriched immune architecture through compartment-specific spatial associations with CD8^+^ cytotoxic T cells, regulatory T cells, and checkpoint-expressing populations, forming a coordinated effector-regulatory niche unique to the invasive margin.

Taken together, these results demonstrate that high CD8/PD-L1 colorectal tumors are characterized by a spatially organized and compartment-specific immune architecture concentrated at the IM compared to wherein myeloid-derived PD-L1^+^ cells establish an immunoregulatory interface with coordinated effector populations (CD3^+^/CD8^+^ T lymphocytes, B cells, NK cells) and regulatory subsets (PD-L1^+^ cells, PD-1^+^ and CD8^+^PD-1^+^ checkpoint expressing cells) alongside IM-enriched Treg cells, defining a discrete immunological phenotype with potential implications for therapeutic response (**Figure 5E**).

## 4 Discussion

CRC is one of the major causes of cancer mortality globally (1). Although classic diagnostic criteria such as TNM staging and MMR status have proven to be gold standard for prognostic and therapeutic decisions, the immune heterogeneity that informs therapeutic outcomes in CRC is lacking (21, 22). The Immunoscore, measuring the CD3^+^ and CD8^+^ cell densities at the TC and IM, has significantly improved risk stratification and prognostic assessment (23). However, its value for predicting clinical benefit from ICI is limited, particularly outside the MSI-H tumors (24). To address this limitation, the Immunoscore-IC classifier was developed by integrating both CD8^+^ and PD-L1^+^ spatial features within the TC (11). While this composite immune metric has improved the prediction of ICI benefit, the biological basis and spatial context of these immune signals remain incompletely understood. We therefore assessed CD8 and PD-L1 densities and proximity in the IM and TC, stratified patients by their combined infiltration status across both compartments. We comprehensively profiled the tumor immune microenvironment and identified spatially distinct effector and suppressive niches in both IM and TC that differ between high and low CD8/PD-L1 infiltration phenotypes.

Our findings demonstrate the context-dependent PD-L1 biology in CRC, a predominantly tumor-intrinsic marker of adaptive resistance (25, 26), PD-L1 in CRC was predominantly derived from stromal and immune compartments, with significant enrichment in CD163^+^ macrophages at the IM rather than tumor epithelium. This pattern aligns with reports that stromal PD-L1^+^ myeloid cells accumulate at the invasive front in MSI-H CRC and can constrain T-cell activity, helping explain why tumor-cell PD-L1 staining alone has limited predictive value for ICI response in CRC (27, 28). These results justify the development of spatially dependent biomarkers based on cellular source and anatomic position of PD-L1.

The IM emerges as a critical site of tumor-immune interaction, where T cells concentrate yet encounter local immunoregulatory programs that attenuate antitumor function (8, 29). Our spatial analysis revealed that PD-L1^+^ cells and CD163^+^ macrophages were co-enriched and positively correlated within the IM, indicating a dominant role for myeloid populations in regional immune suppression. These tumor-associated macrophages (TAMs) have been implicated in inhibiting effector T-cell function, regulating cytokine network, and remodeling the stroma architecture (30). The observed spatial proximity between PD-L1^+^ and CD163^+^ cells support the notion that macrophage-mediated PD-L1 signaling may dampen T-cell–driven antitumor immunity. Therapeutic strategies targeting macrophage function, such as CSF1R inhibitors, CD40 agonists, and TGF-β blockade, may therefore be rational partners for ICIs-based treatment in CRC (31–35).

Spatial profiling revealed a complex IM microenvironment consisting of two functionally distinct yet spatially coordinated niches. An immunoregulatory niche was characterized by co-enrichment of PD-L1^+^ myeloid cells, PD-1^+^ and CD8^+^PD-1^+^ checkpoint-expressing T cells, and CD56^+^ NK cells in high CD8/PD-L1 tumors. Building on the observed correlation between CD4^+^Foxp3^+^ Treg cells and PD-L1^+^ cells across tumor compartments, spatial analysis revealed that Treg cells demonstrated IM-specific positive correlations with PD-1^+^, CD8^+^PD-1^+^, and CD56^+^ NK cells, indicating coordinated regulatory architecture. This spatial organization has important therapeutic implications. NK cells can participate in Treg depletion through anti-CTLA-4–mediated antibody-dependent cellular cytotoxicity (ADCC), providing the mechanistic rationale for Fc-enhanced anti-CTLA-4 combination strategies in pMMR/MSS CRC (36). These spatial relationships appeared more informative for immune dysfunction and therapeutic response than cell abundance alone (13, 20). In contrast, an immune activation niche featured coordinated enrichment of effector populations including CD8^+^ and CD3^+^ T lymphocytes, CD56^+^ NK cells, and CD20^+^ B cells. CD3^+^ and CD8^+^ lymphocyte densities, as defined in the Immunoscore framework, differentiate “hot” from “cold” tumors and provide prognostic information (37). NK cells contribute to anti-tumor immunity through DC recruitment and T-cell activation, supporting favorable clinical outcomes (23, 38, 39). Additionally, B cells, as principal components of tertiary lymphoid structures (TLS), induce potent antitumor immunity and associate with extended survival (40). These findings suggest that the IM serves as a critical spatial interface where immune activation and regulatory programs are co-localized.

Recent studies have led to the expansion of the established paradigm through the identification of an interferon (IFN)-rich immunophenotype, characterized by a substantial presence of CD8^+^ cytotoxic T lymphocytes (CTLs) and antigen-presenting macrophages. This immunophenotype has been identified as a mediator of the efficacy of ICIs in both dMMR/MSI-H and pMMR/MSS CRC subsets (4, 41). CD8^+^ IFN cells can induce MHC class II and CD74 expression in neighboring tumors and myeloid cells, thereby promoting antigen presentation. Increased CD74 is linked to prolonged progression-free survival (PFS), as well as increased responses to ICIs, independent of MMR status or tumor mutational burden (TMB). Continuous IFN signaling, on the other hand, further facilitates adaptive immune resistance through a persistent upregulation of PD-L1 (42, 43). IFN-induced programs are initiated and amplified by myeloid cells and DCs, resulting in feedback loops that further suppress the immune response. Our results reflect the potential of integrating spatial features tied to IFN, such as CD74 expression or the spatial proximity of T cells to macrophages, into analyses of CD8 and PD-L1 infiltration to better guide the biological specificity of immune classification in CRC. Spatially defined immune markers may also be predictive across all tumors including pMMR/MSS CRC, as immune heterogeneity remains extensive. Integrating CD8 and PD-L1 spatial variables into molecular classification systems, such as the Consensus Molecular Subtypes (CMS) and intrinsic CMS (iCMS), can advance immune stratification (44, 45). Considering the established biological distinction associated with primary and metastatic CRC lesions (46, 47), the spatial organization of CD8 and PD-L1 in liver and lung metastases is important for therapeutic decision-making.

This exploratory study reveals promising observations regarding spatial organization of immune cells in the CRC microenvironment. While our findings are robust within this limited cohort, the relatively small sample size limits statistical power. These observations should be considered hypothesis-generating and require validation in larger, independent multicenter cohorts. External validation presents methodological challenges. Bulk transcriptomic repositories such as TCGA lack the spatial resolution required to interrogate cell-cell proximity dynamics. Single-marker immunohistochemistry cannot recapitulate the multi-dimensional spatial phenotypes identified here, and spatial transcriptomics, while orthogonal, measures distinct biological features from protein-based multiplex immunofluorescence. Notably, our observations align with prior reports demonstrating that T-cell suppression in CRC correlates with spatial proximity between PD-L1^+^ and PD-1^+^ cells (13, 20). While proximity of PD-L1^+^ macrophages to PD-1^+^ T cells indicates potential functional communication based on spatial co-localization, definitive evidence of such interactions depends on complementary approaches including spatial transcriptomics, high-dimensional imaging, and single-cell multi-omics to provide direct information on ligand–receptor dynamics and cell state transitions *in situ* (48). Longitudinal sampling during therapy regimens would further contribute to understanding temporal variability and treatment-induced immune remodeling dynamics.

In summary, this study establishes that CRC demonstrates a distinctive non-tumor-centric PD-L1 pattern, predominantly expressed by CD163^+^ macrophages at the IM. High CD8/PD-L1 tumors are characterized by spatially organized IM niches where coordinated effector and regulatory populations coexist, with IM-specific Treg correlations providing rationale for combined checkpoint blockade. These findings demonstrate that comprehensive spatial profiling of IM-enriched populations alongside CD8/PD-L1 could refine patient stratification and guide selection of combination therapies targeting these populations with checkpoint blockade in CRC.

## Data Availability

The raw data supporting the conclusions of this article will be made available by the authors, without undue reservation.
