# Supplementary material for: Myeloid-derived PD-L1 characterizes spatially organized immune architecture in colorectal cancer
Source: Front Immunol. 2026 Feb 6;17:1763068. doi: 10.3389/fimmu.2026.1763068 (PMC12920468; doi:10.3389/fimmu.2026.1763068)
Supplement: Supplementary file 1 [file DataSheet1.pdf]

Supplementary Material

1 Supplementary Tables and Figures

1.1 Supplementary Tables

1.1.1 Table S1 The characteristics of primary CRC samples for mIF staining

**Characteristic**	**N (%)**
Total patients	15
Gender	
Female	9 (60%)
Male	6 (40%)
Age	
> 60 years	4 (26.7%)
< 60 years	11 (73.3%)
Tumor stage	
I+II	8 (53.3%)
III+IV	7 (46.7%)
Tumor locations	
Left	9 (60%)
Right	6 (40%)
Molecular Subtypes	
MSS	7 (46.7%)
MSI-H	8 (53.3%)

1.2 Supplementary Figures

1.2.1 **Figure S1** mIF panels and quantification of CD8/PD-L1 features

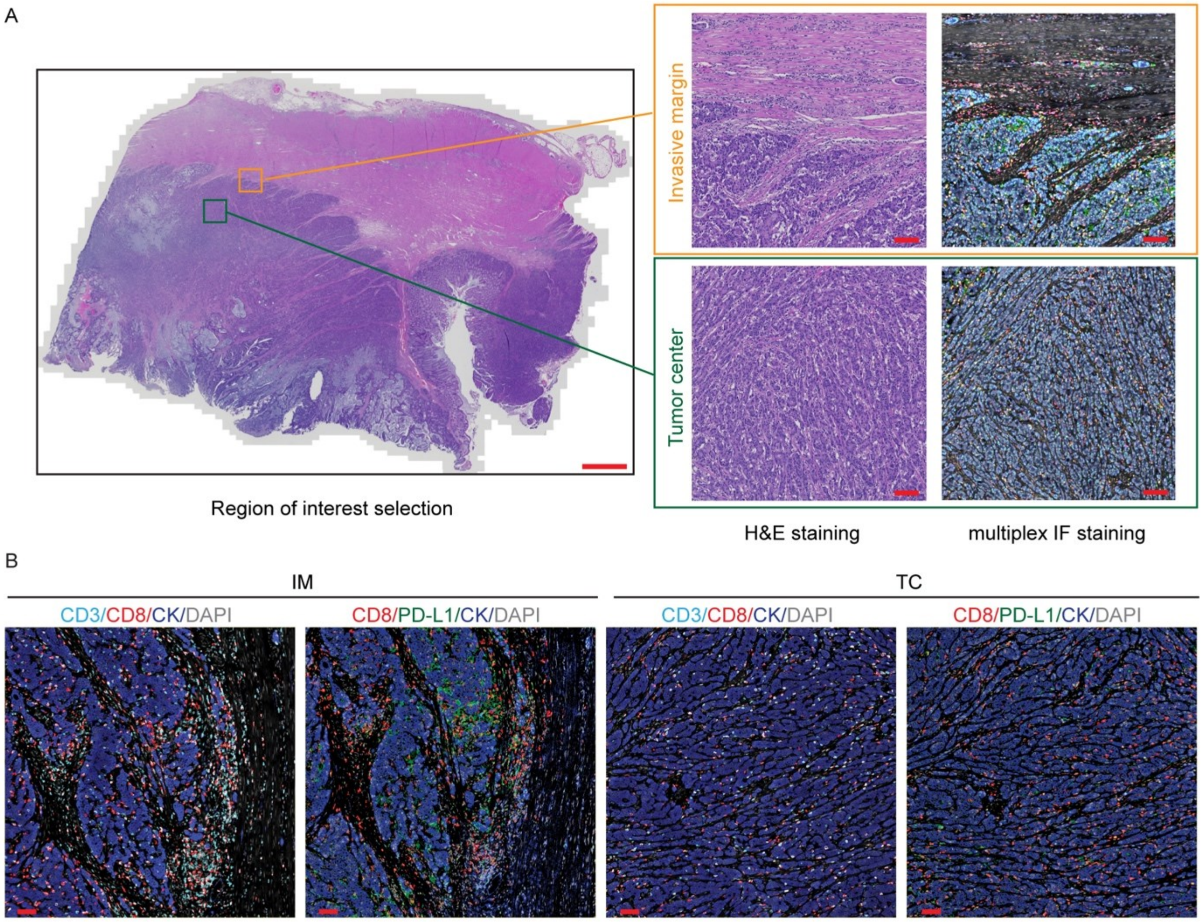

(**A**) Illustration of tumor center (TC) and invasive margin (IM) selection on mIF images, guided by morphology on H&E-stained whole-slide images. Scale bar: 2 mm (left); 100 μm (right). H&E, hematoxylin and eosin. IF, immunofluorescence. (**B**) Representative mIF images showing CD3, CD8, and PD-L1 distributions in IM and TC. Scale bar: 100 μm

1.2.2 **Figure S2** PD-L1 expression patterns in CRC

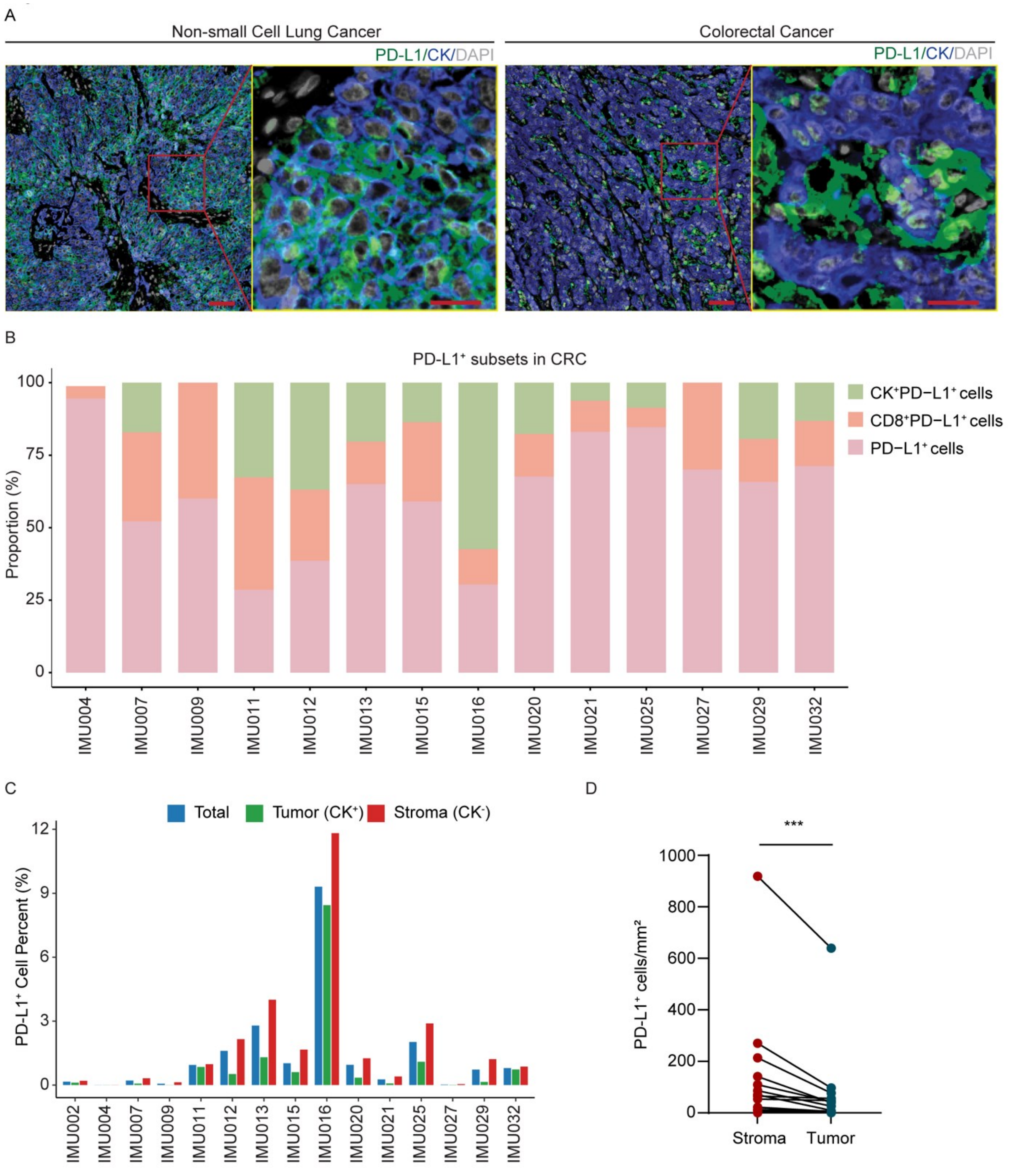

(**A**) Representative PD-L1 staining patterns in NSCLC versus CRC; scale bar. Scale bar: 50 μm (**B**) Composition of PD-L1^{+} subtypes in CRC from mIF Panel 2, categorizing PD-L1–expressing cells as single PD-L1^{+}, CK^{+}PD-L1^{+} (co-localized with CK^{+} tumor cells), or CD8^{+}PD-L1^{+} (co-localized with CD8^{+} T cells). (**C**) Distribution of PD-L1^{+} cells across total, tumor (CK^{+}), and stromal (CK^{-})

compartments. (**D**) Paired analysis showing significantly higher PD-L1^{+} cell density in stroma versus tumor. ns, $p > 0.05$; $***p < 0.001$.

1.2.3 **Figure S3** Spatial relationships of immune cell subsets in CRC

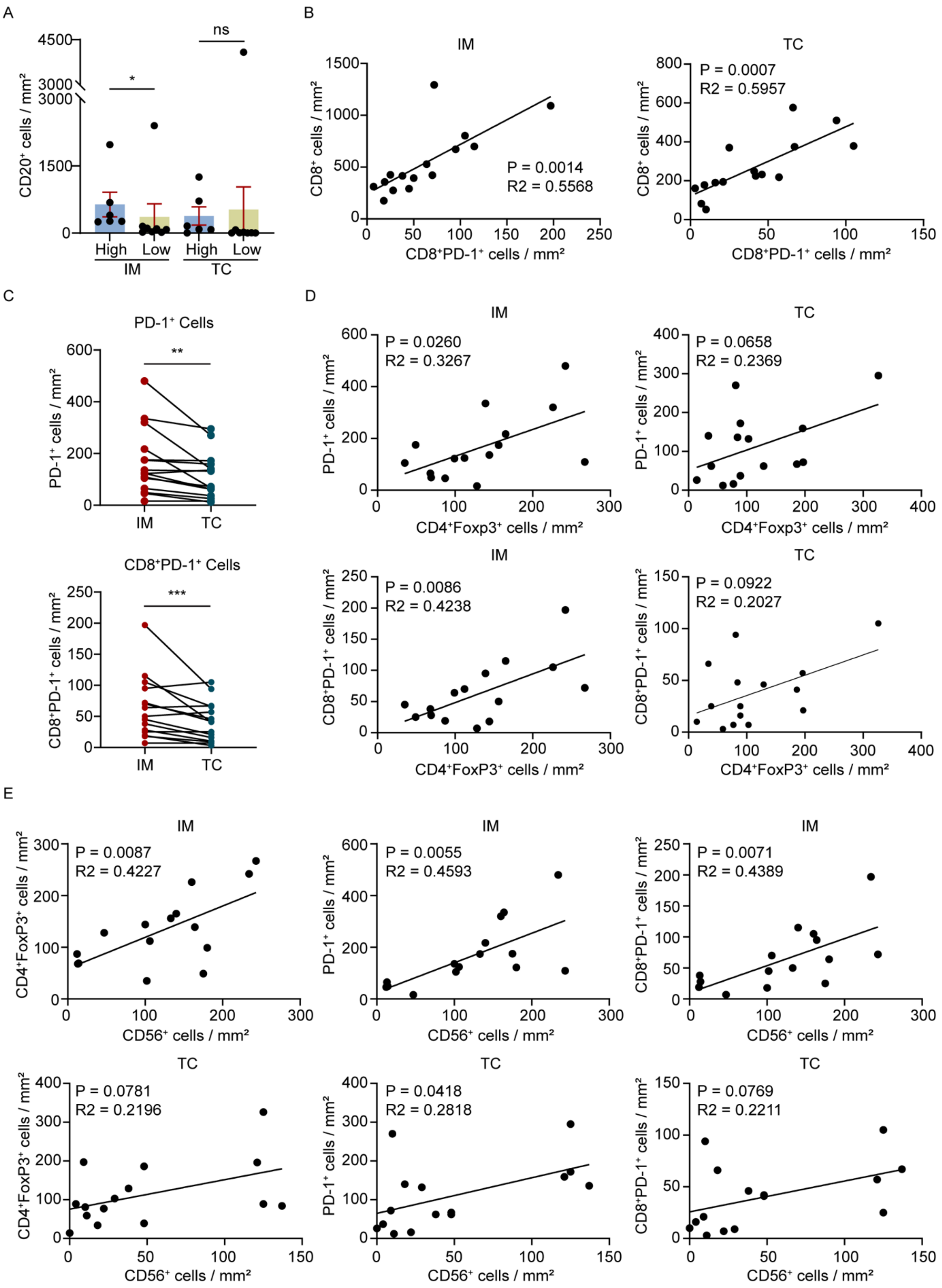

(A) CD20$^+$ cell densities in the IM and TC across CD8/PD-L1 infiltration groups. (**B**) Correlations between CD8$^+$ and CD8$^+$PD-1$^+$ cell densities in CRC with different regions (IM and TC). (**C**) Paired comparison of PD-1$^+$ and CD8$^+$PD-1$^+$ cell densities between IM and TC using the Wilcoxon matched-pairs signed-rank test (n = 15). (**D**) Correlations between CD4$^+$Foxp3$^+$ cells and CD8$^+$ or CD8$^+$PD-1$^+$ cell densities in CRC with different regions (IM and TC). (**E**) Correlations between CD56$^+$ NK cell densities and CD4$^+$Foxp3$^+$ Tregs, CD8$^+$, or CD8$^+$PD-1$^+$ cell densities in CRC with different regions (IM and TC). ns, $p > 0.05$; $*p \leq 0.05$; $**p < 0.01$; $***p < 0.001$.
